# State of Wisconsin

**Published:** 1891-03

**Authors:** V. T. Atkinson

**Affiliations:** State Veterinarian


### STATE OF WISCONSIN.

The following letter has been issued to all veterinary graduates in Wisconsin, so far as their names were known.

February 20, 1891.

DEAR SIR :

After conferring with many of the veterinary graduates of the State, we have reached the conclusion that the time has now arrived for the formation of an association to be composed of graduates only. With this purpose in view you are invited to meet with us at Madison, on the 18th of March. Our headquarters will be at the rooms of the State Agricultural Society in the Capitol.

Invite any *regular graduate* to come with you.

Cordially yours,

V. T. ATKINSON,

*State Veterinarian.*

